# Protein Stability of Pyruvate Kinase Isozyme M2 Is Mediated by HAUSP

**DOI:** 10.3390/cancers12061548

**Published:** 2020-06-12

**Authors:** Hae-Seul Choi, Chang-Zhu Pei, Jun-Hyeok Park, Soo-Yeon Kim, Seung-Yeon Song, Gyeong-Jin Shin, Kwang-Hyun Baek

**Affiliations:** Department of Biomedical Science, CHA University, Gyeonggi-Do 13488, Korea; chlgotmf01@naver.com (H.-S.C.); czpei1983@163.com (C.-Z.P.); nymwns1@naver.com (J.-H.P.); ssyy217@naver.com (S.-Y.K.); seungyon95@naver.com (S.-Y.S.); mu6368@daum.net (G.-J.S.)

**Keywords:** deubiquitination, protease, ubiquitin, ubiquitination, ubiquitin ligase, ubiquitin-specific protease 7, UPS

## Abstract

The ubiquitin–proteasome system (UPS) is responsible for proteasomal degradation, regulating the half-life of the protein. Deubiquitinating enzymes (DUBs) are components of the UPS and inhibit degradation by removing ubiquitins from protein substrates. Herpesvirus-associated ubiquitin-specific protease (HAUSP) is one such deubiquitinating enzyme and has been closely associated with tumor development. In a previous study, we isolated putative HAUSP binding substrates by two-dimensional electrophoresis (2-DE) and identified them by matrix-assisted laser desorption-ionization time-of-flight mass spectrometry (MALDI-TOF/MS) analysis. The analysis showed that pyruvate kinase isoenzyme M2 (PKM2) was likely to be one of the substrates for HAUSP. Further study revealed that PKM2 binds to HAUSP, confirming the interaction between these proteins, and that PKM2 possesses the putative HAUSP binding motif, E or P/AXXS. Therefore, we generated mutant forms of PKM2 S57A, S97A, and S346A, and found that S57A had less binding affinity. In a previous study, we demonstrated that PKM2 is regulated by the UPS, and that HAUSP- as a DUB-acted on PKM2, thus siRNA for *HAUSP* increases PKM2 ubiquitination. Our present study newly highlights the direct interaction between HAUSP and PKM2.

## 1. Introduction

Most proteins in eukaryotic cells are degraded by the ubiquitin–proteasome system (UPS). Ubiquitination in this system is mediated by the continuous activities of E1, E2, and E3 enzymes. In addition to proteasomal degradation, single or multiple polyubiquitination can enable proteins to acquire cellular functions [1]. Thus, ubiquitination is a type of post-translational modification (PTM), whereby ubiquitin binds to a target protein and regulates its proteasomal degradation or cellular functions [2]. Ubiquitin contains seven lysine residues (K6, K11, K27, K29, K33, K48, and K63) that can form a polyubiquitin chain; K48 and K63 are the most well-known polyubiquitination sites [3,4,5]. Deubiquitination is an inverse process of ubiquitination. Deubiquitinating enzymes (DUBs) play an essential role in protein stabilization by removing ubiquitins from protein targets [6,7]. To date, approximately 100 DUBs have been identified, and they have shown to be involved in a variety of cellular functions due to their abilities to stabilize or alter the functions of target proteins [8]. The DUB family is composed of at least seven classes. In particular, the cysteine protease class contains ubiquitin-specific protease (USP), ovarian tumor protease (OTU), ubiquitin C-terminal hydrolase (UCH), permutated papain fold peptidases of dsDNA viruses and eukaryote (PPPDE), Machado–Joseph disease protease (MJD), and motif interacting with Ub-containing novel DUB (MINDY) family. The metalloprotease class is represented by the Jab1/Pab1/MPN metalloenzyme motif protease (JAMM) family [9,10]. Herpes virus-associated ubiquitin-specific protease (HAUSP, also known as ubiquitin-specific protease 7) is a cysteine isopeptidase of the USP family [11]. HAUSP is known to be associated with the deubiquitination of p53-related complexes and to regulate cell growth and apoptosis [12,13]. In addition, it has been shown that amino acid sequence E or P/AXXS is a binding motif for HAUSP [14,15]. In a previous study, we identified putative HAUSP substrates by two-dimension electrophoresis (2-DE) and matrix-assisted laser desorption-ionization time-of-flight mass spectrometry (MALDI-TOF/MS) analysis. A proteomics analysis revealed that pyruvate kinase isoenzyme M2 (PKM2) has a high score to be post-translationally modified by HAUSP [16,17]. Under normal conditions, PKM2 acts as a pyruvate kinase in a tetrameric form, but in cancer cells it acts as a protein kinase in a dimeric form [18]. It is known that the UPS regulates PKM2 [19]. Recent experimental studies have shown that PKM2 functions are also related to DUBs or E3 ligases. We confirmed PKM2 ubiquitination and the binding affinity of mutant forms of PKM2. Furthermore, the expression of PKM2 dimers interferes with metabolism and induces the Warburg effect; that is, PKM2 induces anaerobic glycolysis, which is the major process responsible for energy production in most cancer cells [20]. In view of the functions of HAUSP and PKM2, they are likely to be related. In order to investigate their functions, we performed glutathione S-transferase (GST) pull-down, immunocytochemistry, phosphate, ubiquitination, and deubiquitination assays.

## 2. Results

### 2.1. HAUSP Binds to PKM2

**Figure 1:**
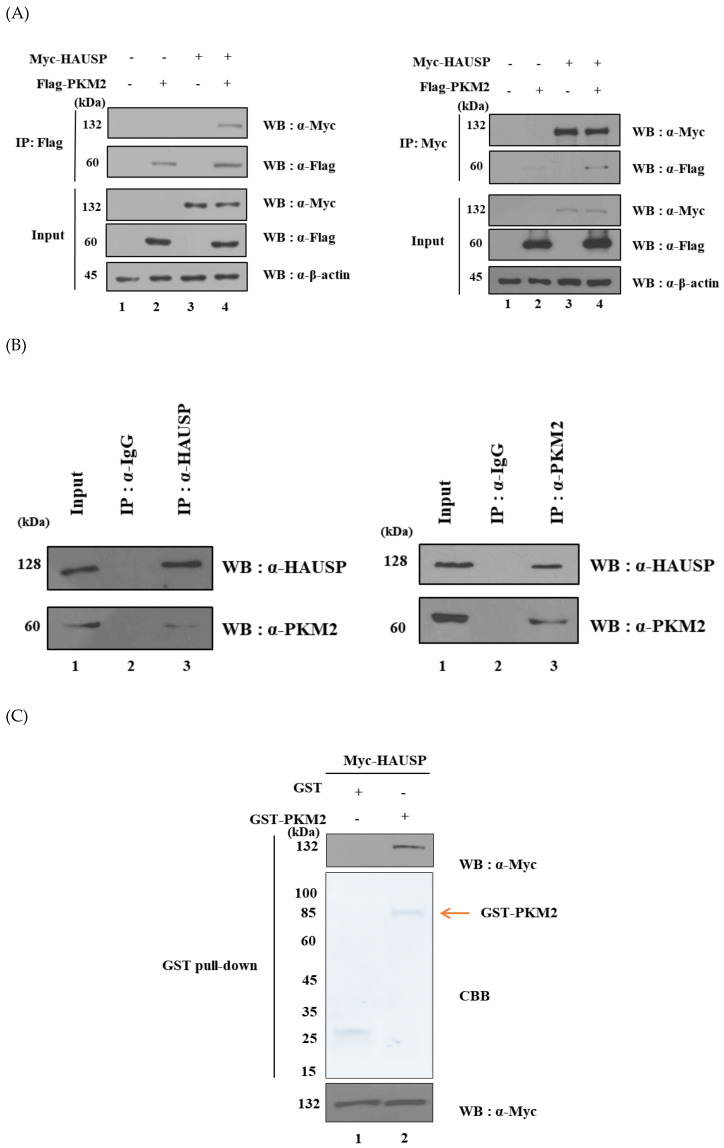

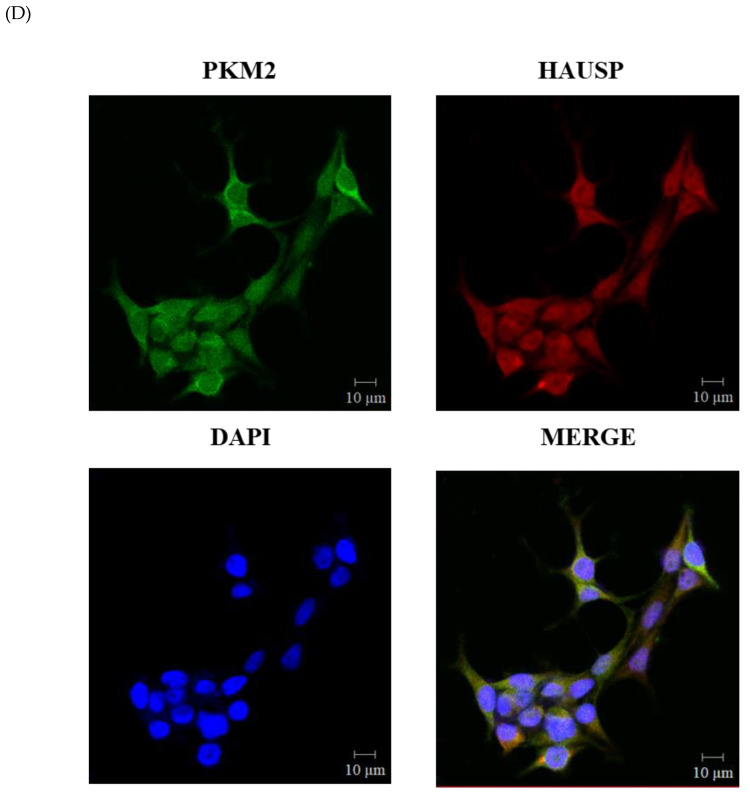
HAUSP binds to PKM2. (**A**) Myc-*HAUSP* and Flag-*PKM2* were co-transfected into HEK293T cells to investigate exogenous binding between Myc-HAUSP and Flag-PKM2. (**B**) HEK293T cell lysates were immunoprecipitated with an anti-HAUSP or an anti-PKM2 antibody to investigate endogenous binding between HAUSP and PKM2. (**C**) GST pull-down assay: GST-PKM2 proteins were obtained from *Escherichia coli* BL21 cells to confirm that PKM2 directly binds to HAUSP. (**D**) Immunocytochemical analysis revealed the nuclear and cytoplasm co-localization of HAUSP and PKM2 in HEK293T cells. Detailed information about western blotting can be found in Figure S1.

In a previous study, we found putative HAUSP binding substrates by 2-DE and MALDI-TOF/MS analysis and PKM2 was identified as one of the putative HAUSP binding substrates [16]. To confirm the interaction between these two proteins, HEK293T cells were co-transfected with Myc-*HAUSP* and Flag-*PKM2* and immunoprecipitated with either an anti-Myc or an anti-Flag antibody. We found that exogenously expressed Myc-HAUSP bound to Flag-PKM2 (Figure 1A). We also performed immunoprecipitation analysis using either an anti-HAUSP or an anti-PKM2 antibody. The results revealed endogenous binding between HAUSP and PKM2 (Figure 1B). In addition, we performed a GST pull-down assay using GST-PKM2 fusion protein to determine whether PKM2 directly binds to HAUSP. GST-PKM2 was incubated with HEK293T whole cell lysates overexpressing Myc-HAUSP and was analyzed by western blotting. The results showed that GST-PKM2 bound directly to Myc-HAUSP (Figure 1C).

### 2.2. Co-Localization of PKM2 and HAUSP

In a previous study, we found that HAUSP was present in the nuclei and cytoplasm of HeLa cells [16]. In the present study, immunocytochemical analysis revealed that HAUSP and PKM2 are co-localized in the nuclei and cytoplasm of HEK293T cells, and DAPI is localized to the nuclei (Figure 1D), which suggests an interaction between HAUSP and PKM2.

### 2.3. Binding Affinity between HAUSP and PKM2

**Figure 2:**
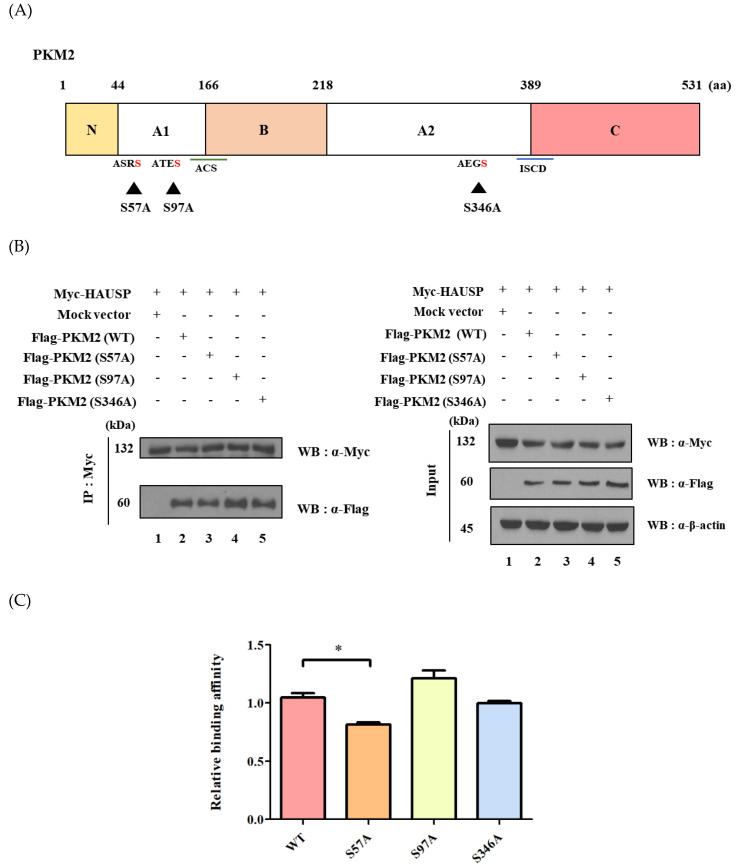
Binding affinity between PKM2 mutants and HAUSP. (**A**) Schematic description of site-directed mutagenesis for *PKM2.* The catalytic active site (ACS) is located between the A1 and B domains of PKM2, and the intersubunit contact domain (ISCD) involved in the formation of tetrameric oligomers is located between the A2 and C domains. The C domain contains the allosteric activator (FBP) binding site and a nuclear localization signal sequence (NLS). N and C are the N-terminal and C-terminal domains, respectively [21]. The putative HAUSP binding motifs are ASRS, ATES, and AEGS. (**B**) HEK293T cells were both transfected with Myc-*HAUSP* and three different Flag-*PKM2* mutant forms. Western blotting for HAUSP and mutant forms of PKM2 was performed. (**C**) Protein levels were determined using three separate experiments. (*n* = *3*, * *p* < 0.05). Detailed information about western blotting can be found in Figure S2.

The amino acid sequence P/AXXS is known to act as a binding motif for HAUSP [14,15]. We found that PKM2 has three HAUSP binding motifs; therefore, we reasoned the ability of HAUSP to bind PKM2 due to the P/AXXS sequence in the A1 and A2 regions of PKM2 (Figure 2A). Binding assays were performed using wild-type and site-directed serine to alanine mutants (S57A, S97A, and S346A of PKM2). These assays revealed that binding affinity of the S57A mutant was less than that of wild-type and other mutants of PKM2. Protein levels of Myc-tagged HAUSP and Flag-tagged PKM2 were expressed differently in HEK293T cells transfected with S57A (Figure 2B,C). It is estimated that the A1 domain, containing S57A, has an effective factor that decreases binding affinity.

### 2.4. PKM2 Protein Level Regulated by HAUSP

**Figure 3:**
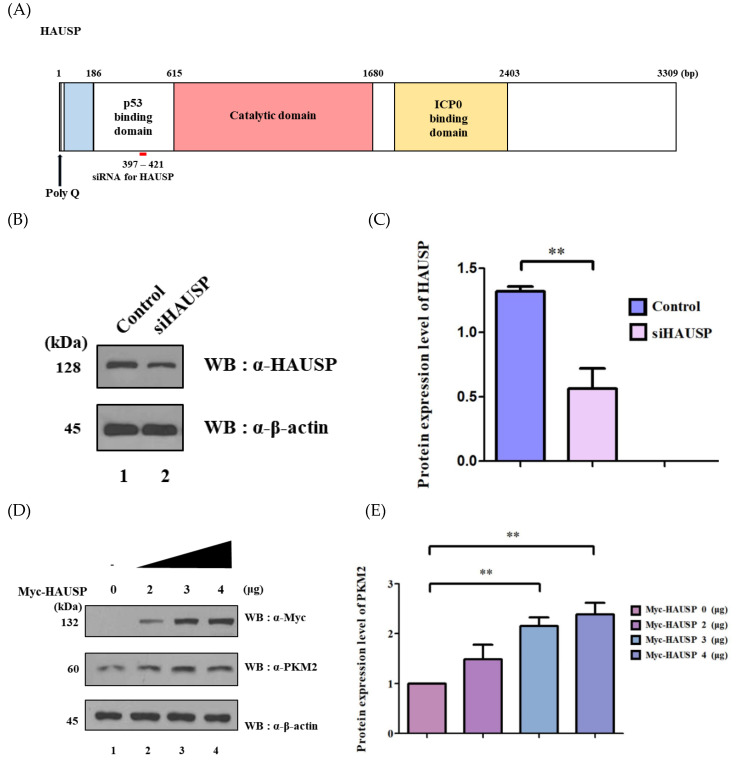

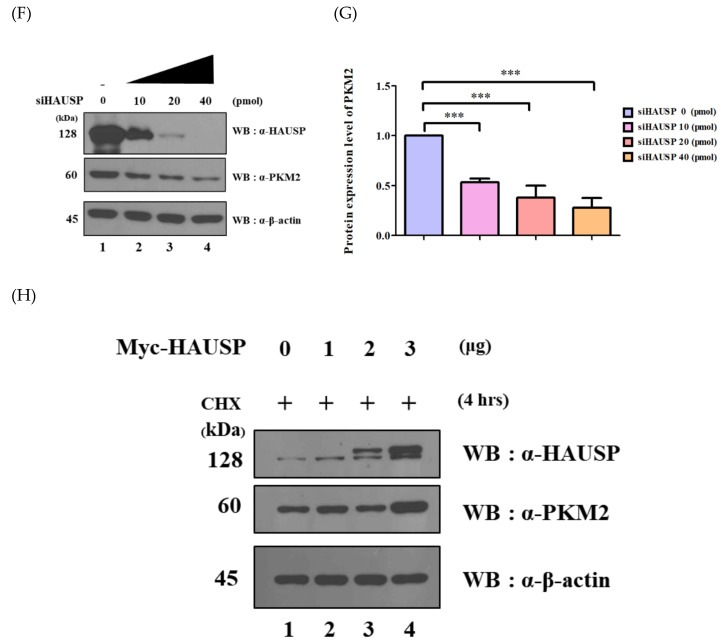
Knockdown effect of HAUSP and dose-dependent regulation of PKM2 in Myc-*HAUSP* or si*HAUSP*-transfected cells. (**A**) Schematic description of *HAUSP* siRNAs. (**B**) HEK293T cells were transfected with 10 pmol si*HAUSP*. (**C**) Knockdown effect of *HAUSP* siRNAs on the expression level of HAUSP protein in HEK293T cells. Protein levels were determined using three separate experiments. (*n* = *3*, ** *p* < 0.01). (**D**) HEK293T and HeLa cells were transfected with different amounts of Myc-*HAUSP* (0, 2, 3, and 4 μg). (**E**) Protein levels were determined using three separate experiments. (*n* = *3*, ** *p* < 0.01). (**F**) HEK293T and HeLa cells were transfected with different amounts of si*HAUSP* (0, 10, 20, or 40 pmol). (**G**) Protein levels were determined using three separate experiments. (*n* = *3*, *** *p* < 0.001). (**H**) CHX assay was performed for checking a half-life of PKM2 and HAUSP in HEK293T cells and western blotting was performed using cell lysates. Detailed information about western blotting can be found in Figure S3.

*HAUSP* siRNA (si*HAUSP*) was designed (Figure 3A) and used as previously described [11]. We first checked knockdown efficiency of si*HAUSP* (Figure 3B). Protein levels were determined using three separate experiments (*n* = *3*, ** *p* < 0.01) (Figure 3C). Then, with the expression of Myc-HAUSP in a dose-dependent manner, we found that PKM2 gradually increased (Figure 3D,E). As expected, we found that the protein level of PKM2 decreased in a dose-dependent manner of si*HAUSP* (Figure 3F,G). In addition, cycloheximide (CHX) assay was carried out to investigate the half-life of PKM2. As HAUSP was expressed in a dose-dependent manner, the protein level of PKM2 increased (Figure 3H). These observations confirmed that HAUSP regulates the half-life of PKM2.

### 2.5. Differential Ubiquitnation of PKM2 Mediated by the Enzymatic Activity of HAUSP

**Figure 4:**
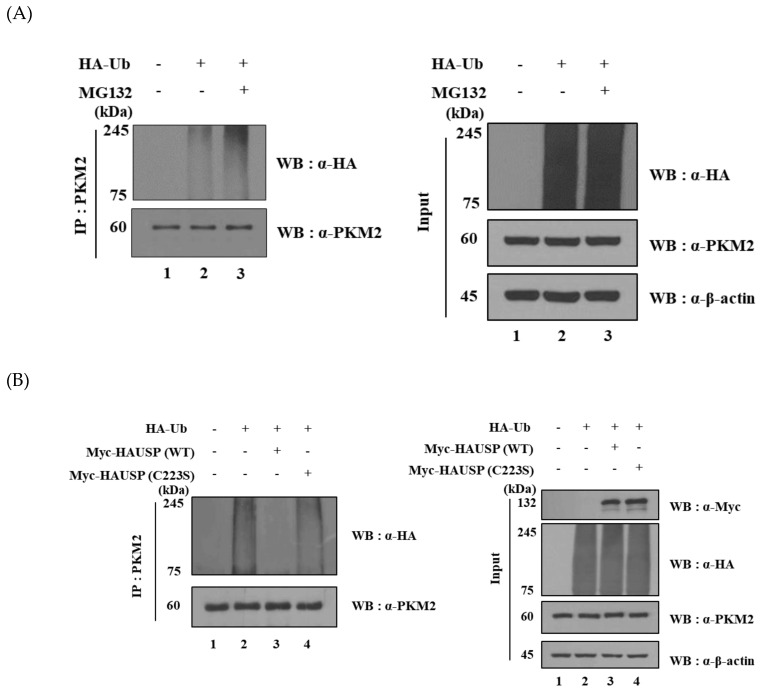

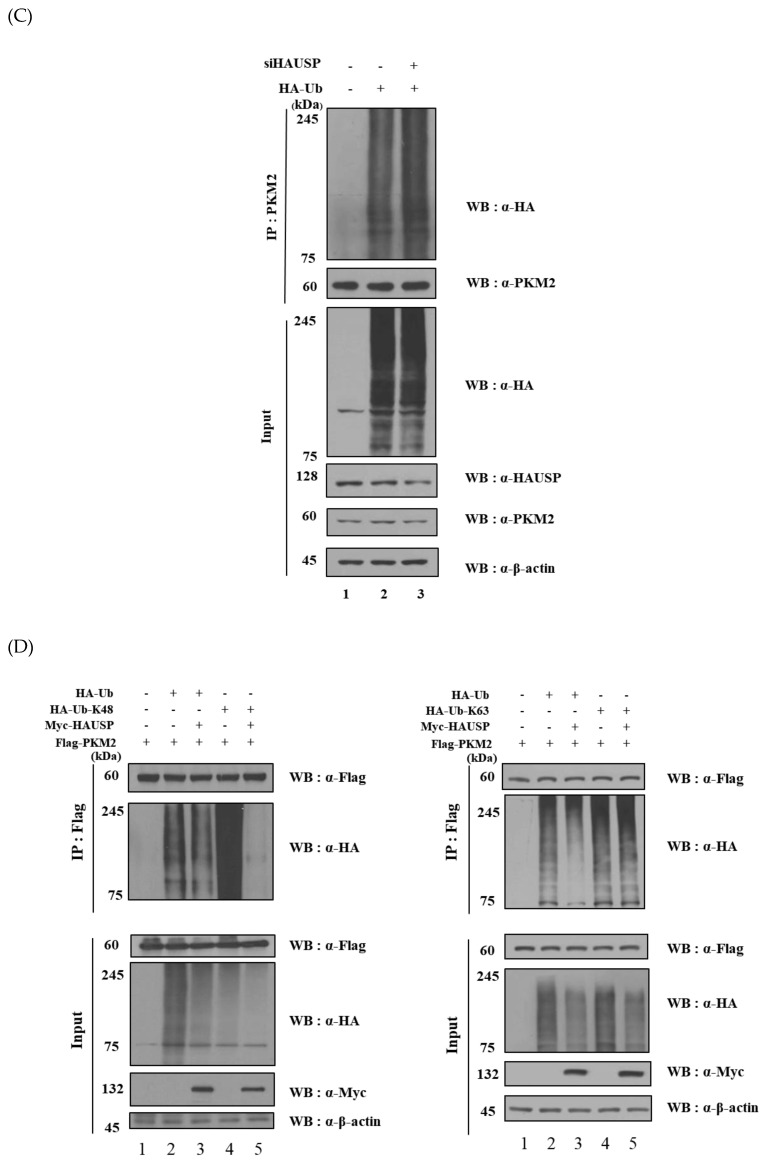
Differential ubiquitination of PKM2 regulated by the enzymatic activity of HAUSP. (**A**) Protein levels of PKM2 in HEK293T cells are regulated by the UPS. (**B**) Deubiquitination levels in the presence of wild-type or a catalytically inactive form of HAUSP. (**C**) Ubiquitination assay for HAUSP downregulated with siRNA treatment. Immunoprecipitation was performed using an anti-PKM2 antibody. (**D**) For deubiquitination assay, HEK293T cells were transfected with Flag-*PKM2* or Myc-*HAUSP* along with HA-*Ub* or HA-*Ub*-K48 or HA-*Ub*-K63. Immunoprecipitation was performed by an anti-Flag antibody. Detailed information about western blotting can be found in Figure S4.

We performed ubiquitination assays to determine whether PKM2 is regulated by the UPS. To determine whether PKM2 undergoes ubiquitination, HEK293T cells were transfected with HA-*ubiquitin*, and then PKM2 ubiquitination assays were performed. The results obtained showed that the ubiquitination level of PKM2 was increased by the treatment of a proteasome inhibitor MG132, indicating that PKM2 is regulated by the UPS. Cell lysates were subjected to immunoprecipitation using anti-HA and anti-PKM2 antibodies (Figure 4A).

### 2.6. HAUSP Has DUB Activity Towards PKM2

To confirm that deubiquitination of PKM2 is mediated by HAUSP, deubiquitination assays were performed using Myc*-HAUSP* (WT) and a catalytically inactive mutant of Myc-*HAUSP* (C223S). The ubiquitination of PKM2 was reduced by Myc-*HAUSP*, but not by Myc-*HAUSP* (C223S). This result indicates that HAUSP can remove ubiquitin from PKM2 (Figure 4B). We performed western blotting and immunoprecipitation for checking knockdown of PKM2 protein with si*HAUSP*. When si*HAUSP* was transfected into HEK293T cells, the ubiquitination level of PKM2 increased. Thus, HAUSP has its enzymatic activity towards PKM2 and si*HAUSP* can upregulate the ubiquitination level of PKM2 (Figure 4C). We also confirmed that polyubiquitin chains are assembled through the K48 and K63 of ubiquitin on PKM2. It is of interest that HAUSP has DUB activity for K48 of ubiquitin-linked PKM2 (Figure 4D).

## 3. Discussion

The purpose of this study was to investigate whether HAUSP can interact with and regulate PKM2, which was identified as a putative binding protein of HAUSP in a previous study [16]. Interaction between HAUSP and PKM2 was demonstrated by immunoprecipitation, and GST pull-down assay revealed that these two proteins directly interacted with each other (Figure 1). PKM2 is an isoenzyme of the pyruvate kinase family and catalyzes glycolysis. PKM2 also promotes the phosphorylation of phosphoenolpyruvate, the production of ATP, and cancer cell growth [22,23]. PKM2 benefits cancer cells by inhibiting glycolysis and by allowing carbohydrate metabolisms—including uridine diphosphate (UDP)-glucose synthesis, glycerol synthesis, and the hexosamine pathway—to form macromolecular precursors with other adjuvant pathways [24]. Although it has been suggested that PKM2 may be involved in cancer cell proliferation and tumorigenesis, it remains unclear whether PKM2 is involved in these processes [17,25]. It has also been reported that PKM2 is ubiquitinated through the K48 and K63 of ubiquitin, and the protein level of PKM2 is mediated by USP20 [26]. In humans, HAUSP also interacts with the E3 ligase Trip12, which participates in the regulation of DNA damage response [27]. Phosphorylation of STAT3 at S727 promotes apoptosis, whereas phosphorylation at Y705 inhibits apoptosis by promoting nuclear Bcl-2 (B-cell lymphoma protein 2) expression [28,29]. Furthermore, PKM2 promotes the phosphorylation of STAT3 at Y705 [29]. MDM2 is an important negative regulator of p53, and HAUSP regulates both MDM2 and p53 [11,30]. Catalytic activity of HAUSP may be regulated by diverse HAUSP inhibitors, including P22077 and HBX41108, and interaction between DUBs and p53 has an important impact on the development of targeted therapies for p53-related cancers [31]. HAUSP downregulates Bcl-2 and, thus, invokes apoptosis [32]. In addition, STAT invokes expression of c-Myc and p21 in the cell cycle. Besides, PKM2 functions through Akt phosphorylation [33]. Therefore, both PKM2 and Akt are regulated in the cell metabolic pathway. In a recent study, Akt was shown to increase cancer growth and metabolism via the phosphorylation of PKM2 [22].

In previous study, E or P/AXXS was known as the binding motif of HAUSP [17,18]. Therefore, we investigated whether putative HAUSP binding motifs are present in PKM2. Three putative binding motifs were identified, and S57A, S97A, and S346A mutants in these motifs of PKM2 were generated. It is of interest that the single mutant S57A predominantly affected PKM2 to HAUSP binding affinity. Both S97A and S346A also affected binding affinity to some extent (Figure 2). PKM2 is expressed in proportion to HAUSP expression in HEK293T and HeLa cells (Figure 3). That indicates that HAUSP is a regulator of PKM2. Our deubiquitination assay revealed that it mediates the deubiquitination on K48 of ubiquitin on PKM2 (Figure 3). Further experiments are required to determine the role of HAUSP-PKM2 binding to the development of therapeutics for diseases like cancer. PKM2 contributes to the metabolism in several cancers—especially in renal cell, thyroid, and breast cancers—and promotes breast cancer cells through the Wnt/β-catenin pathway [34,35]. HAUSP is being extensively studied at present and is perhaps the best understood USP. HAUSP is believed to have many roles and functions in humans [16]. PKM2 is expressed in normal embryonic cells, particularly in adipose tissues and retina and in cells that rapidly synthesize nucleic acids, like tumor cells. HAUSP seems to be able to regulate PKM2 in cancer. However, when Myc-*HAUSP* was transfected, or when Flag-*PKM2* and Myc-*HAUSP* were co-transfected into HeLa cells, cells died about 24 h after transfection. In other words, HAUSP upregulation increased the protein level of PKM2, but also caused apoptosis. On the other hand, si*HAUSP* reduced the expression of HAUSP and the protein level of PKM2, suggesting that si*HAUSP* may have therapeutic potential for cancer development. However, further experiments will be significant in determining the role of HAUSP on PKM2 in a physiological condition, such as in-vivo model systems. Taken all together, the interaction between PKM2 and HAUSP has considerable implications for the development of new drugs and biomarkers.

## 4. Materials and Methods

### 4.1. Construction of Expression Vectors and siRNAs

Full-length cDNAs of *HAUSP* (WT), *HAUSP* (C223S), and *PKM2* were used as previously described [11,26]. For GST pull-down assay, *PKM2* was subcloned into pGEX4T-1 plasmid vector. A pcDNA3.1-6-Myc vector was used to subclone *HAUSP*. For exogenous analysis of putative *PKM2* mutant forms of its HAUSP binding motif, primers of S57A: 5′-GCT TCC CGA GCA GTG GAG ACG-3′, S97A: 5′-GCC ACG GAA GCC TTT GCT TCT-3′, and S346A: 5′-GCT GAA GGC GCT GAT GTG GCC-3′ were used for site-directed mutagenesis. The sequence of the si*HAUSP* used was 5′-CAU GCAC AGG CAG UGC UGA AGA UAA-3′ (UbiProtein Corp, Seongnam, Korea). siRNA transfections were performed using Lipofectamine 2000 Reagent (Invitrogen, Waltham, MA, USA). HA-*ubiquitin* (WT, K48, and K63) constructs confirmed in our previous study were used for ubiquitination and deubiquitination assays [36].

### 4.2. Cell Culture Conditions, Transfection, and Antibodies

HEK293T (*ATCC*, Manassas, VA, USA) cells and HeLa cells (*ATCC*, Manassas, VA, USA) were grown in Dulbecco’s Modified Eagle’s Medium (DMEM, Gibco, Grand Island, NY, USA) containing 10% fetal bovine serum (FBS, Gibco, Grand Island, NY, USA), 1% antibiotic-antimycotic reagent (Gibco, Grand Island, NY, USA) at 37 °C in a 5% CO₂ incubator. Transfection with Lipofectamine was performed using Opti-MEM and Lipofectamine2000 (Invitrogen, Carlsbad, CA, USA). Polyethyleneimine (PEI, Polysciences, Inc., Warrington, PA, USA) transfections were performed using 150 mM NaCl and 10 mM polyethyleneimine for 24 to 48 h. Cells were harvested after 20–24 h of transfection with Myc-*HAUSP*. Anti-Flag (Sigma-Aldrich, St. Louis, MO, USA), anti-Myc (9E10 hybridoma cell media), anti-HA (12CA5 hybridoma cell media), anti-HAUSP (sc-30164, Santa Cruz Biotechnology, Santa Cruz, CA, USA), anti-PKM2 (sc-365684, Santa Cruz Biotechnology, CA, USA), and anti-β-actin (sc-47778, Santa Cruz Biotechnology CA, USA) antibodies were used for GST pull-down assays, immunoprecipitation, immunoblotting, and immunocytochemistry.

### 4.3. Western Blotting and Immunoprecipitation

HEK293T cells were lysed with lysis buffer (50 mM Tris-HCl [pH 7.5], 1 mM EDTA, 10% Glycerol, 300 mM NaCl and 1% Triton X-100). Samples were incubated in an ice-cold environment for 20 min and centrifuged at 13,000 rpm for 20 min at 4 °C, and supernatants were collected. Western blotting was performed using SDS gels, and proteins were transferred to polyvinylidene fluoride (PVDF) microporous membranes (Millipore, Billerica, MA, USA), which were then blocked with TTBS (20 mM Tris-HCl [pH 7.5], 0.05% Tween 20 and 150 mM NaCl) containing 5% skim milk for 1 h, and incubated overnight at 4 °C with primary antibodies. Membranes were then washed three times for 7 min each with TTBS, incubated for 1 h with the secondary antibody, and rewashed three more times in TTBS. Blots were detected using ECL reagent solution (Young-In Frontier, Seoul, Korea). For the immunoprecipitation study, cell lysates were incubated with an antibody at 4 °C overnight and then for 2 h with protein A/G PLUS-Agarose Beads (Santa Cruz Biotechnology, Santa Cruz, CA, USA). Samples were boiled in 2× SDS sample buffer for 7 min and detected by western blotting.

### 4.4. GST Pull-Down Assay

For protein induction, *Escherichia coli* BL21 cells transformed with pGEX-4T-1 or pGEX-4T-1-*PKM2* were incubated at 20 °C overnight. LB broth (5 mL) was then added in a 15 mL conical tube. Transfected cells were induced using 0.5 mM isopropyl β-D-1-thiogalactopyranoside (IPTG) (Promega, Madison, WI, USA) and adjusted to an A_600_ of 0.4–0.5. Cells were lysed by sonication and incubated with glutathione-sepharose beads (Pharmacia Biotech, Uppsala, Sweden). Purified proteins were rotated with HEK293T cell lysates overexpressing Myc-HAUSP at 4 °C overnight, and bound proteins were analyzed by western blotting.

### 4.5. Ubiquitination and Deubiquitination Assays

For PKM2 ubiquitination assays, HEK293T cells were transfected with HA-*ubiquitin* in DMEM medium containing 150 mM NaCl and 10 mM PEI (PEI, Polysciences, Warrington, PA, USA), incubated for 48 h, treated with MG132 (Sigma-Aldrich, St. Louis, MO, USA) for 4 h and harvested. Cells were then lysed in lysis buffer. For immunoprecipitation analysis, lysed cells were incubated with an anti-PKM2 antibody (Sigma-Aldrich, St. Louis, MO, USA) at 4 °C overnight. HAUSP deubiquitination assays were performed using Myc-*HAUSP* and Myc-*HAUSP* (C223S). In this experiment, the anti-PKM2 antibody deposited HA-ubiquitin. IP was performed using the above-mentioned procedure. Deubiquitination assays were performed using HA-*ubiquitin* and Myc-*HAUSP.*

### 4.6. Immunocytochemistry

HEK293T cells were seeded on glass coverslips, placed on a 12-well plate, washed briefly with phosphate-buffered saline (PBS), fixed with 4% formaldehyde for 15 min, blocked with PBS containing 1% BSA (Bovine Serum Albumin) for 1 h at room temperature, and treated with primary antibodies (PKM2 and HAUSP) in 1% BSA at 4 °C overnight. Cells were then washed with PBS, incubated with Alexa-Fluor-488-conjugated goat anti-mouse (1:500, Invitrogen, Carlsbad, CA, USA) and with Alexa-Fluor-568-conjugated goat anti-rabbit 1:500 for 1 h at room temperature in the dark, washed with PBS, and stained with DAPI (1 mg/mL stock, 1:1000, Sigma-Aldrich, St. Louis, MO, USA). Samples were visualized under a confocal microscope (Zeiss LSM880, Carl Zeiss Microscopy GmbH, Jena, Germany).

### 4.7. Cycloheximide Assay

HEK293T cells were transfected with HAUSP in a dose-dependent manner. After 44 h of incubation, CHX (1:1000, 100 mg/mL) was treated into HEK293T cells for 4 h. Then western blotting was performed.

### 4.8. Statistical Analysis

Statistical analysis was conducted using the *t*-test and one-way analysis of variance followed by Tukey’s multiple comparisons post hoc tests using GraphPad Prism version 5 (GraphPad Software, La Jolla, CA, USA). At least three separate tests were performed. Densitometric analysis was conducted using Image J software (Version 1.4.3, National Institutes of Health, Bethesda, MD, USA). *p* values of * *p* < 0.05, ** *p* < 0.01, *** *p* < 0.001 were deemed significant.

## 5. Conclusions

PKM2 directly interacts with HAUSP and increases cell proliferation through the TCA cycle in cancer cells. Although HAUSP is well known to induce apoptosis, this study shows that PKM2 is controlled by HAUSP. In conclusion, additional experiments are required to validate that the suppression of PKM2 levels using HAUSP inhibitors or siRNAs has potential possibility to provide an effective cancer treatment.

## Supplementary Materials

The following are available online at https://www.mdpi.com/2072-6694/12/6/1548/s1, Figure S1: Detailed information about western blot in Figure 1, Figure S2: Detailed information about western blot in Figure 2, Figure S3: Detailed information about western blot in Figure 3, Figure S4: Detailed information about western blot in Figure 4.
