# Supplementary material for: Protein Stability of Pyruvate Kinase Isozyme M2 Is Mediated by HAUSP
Source: Cancers (Basel). 2020 Jun 12;12(6):1548. doi: 10.3390/cancers12061548 (PMC7352364; doi:10.3390/cancers12061548)
Supplement: Supplementary file 1 [file cancers-12-01548-s001.pdf]

Supplementary Materials

Protein Stability of Pyruvate Kinase Isozyme M2 Is Mediated by HAUSP

Hae-Seul Choi, Chang-Zhu Pei, Jun-Hyeok Park, Soo-Yeon Kim, Seung-Yeon Song, Gyeong-Jin Shin and Kwang-Hyun Baek

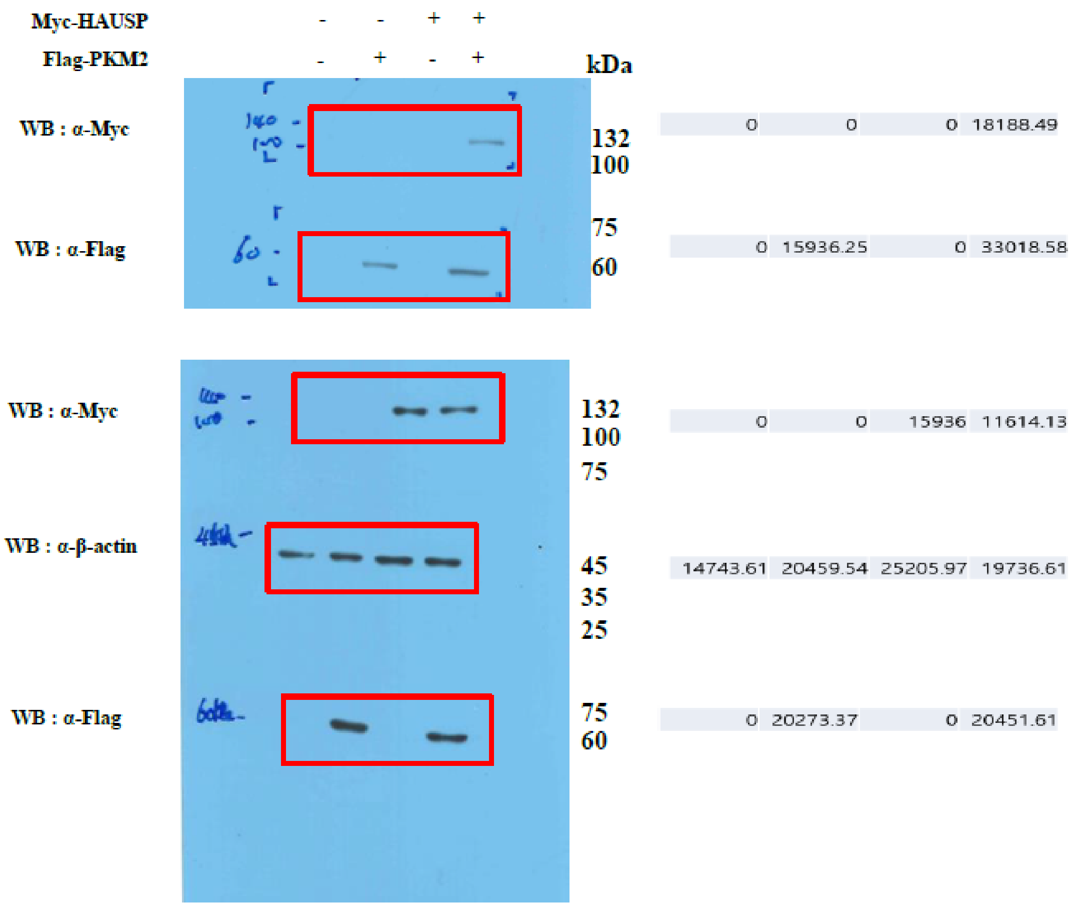

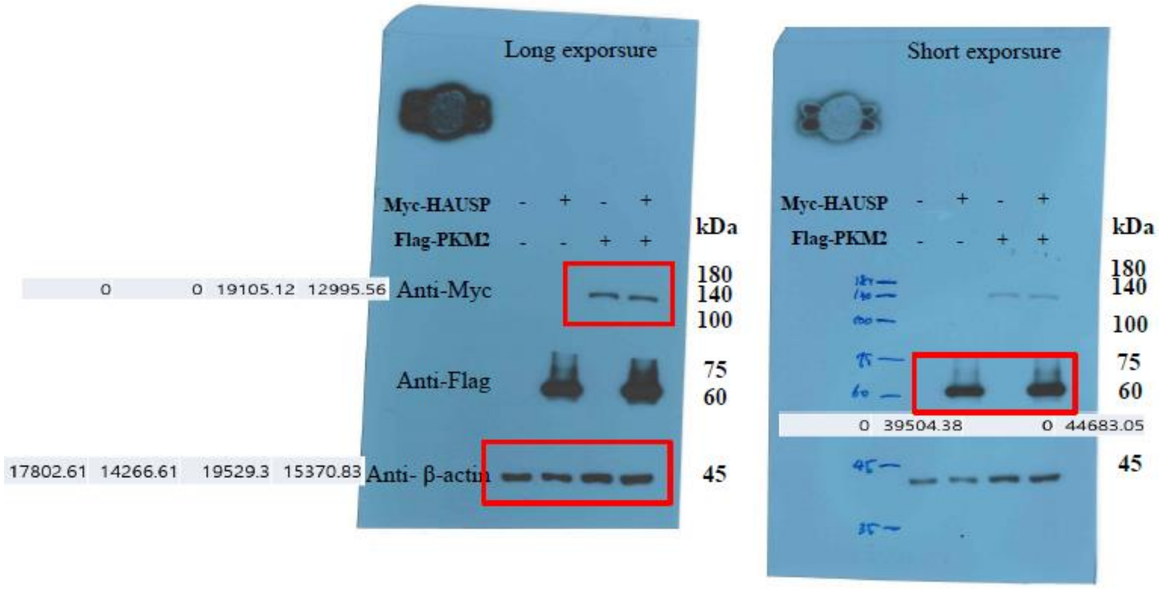

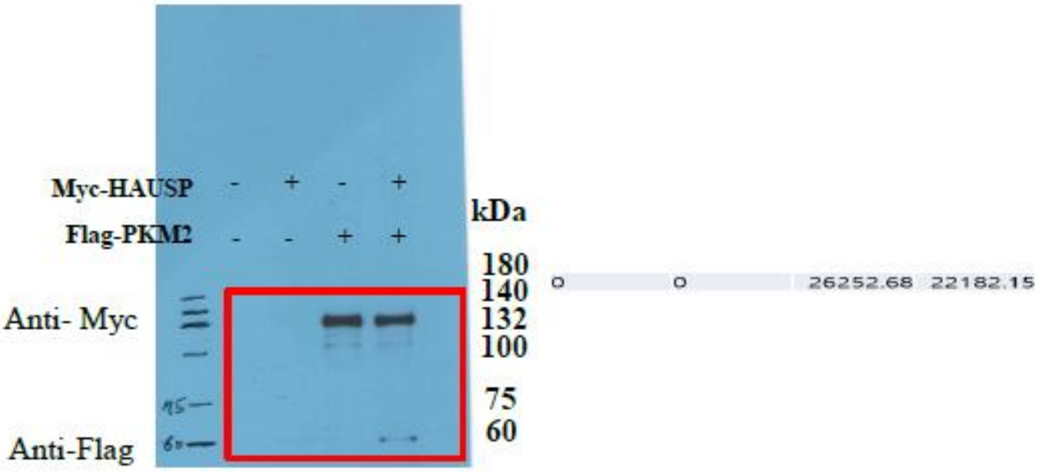
Myc-HAUSP - + - +
Flag-PKM2 - - + +
kDa
180
140
132
100
75
60
Anti- Myc
Anti-Flag
0 0 26252.68 22182.15

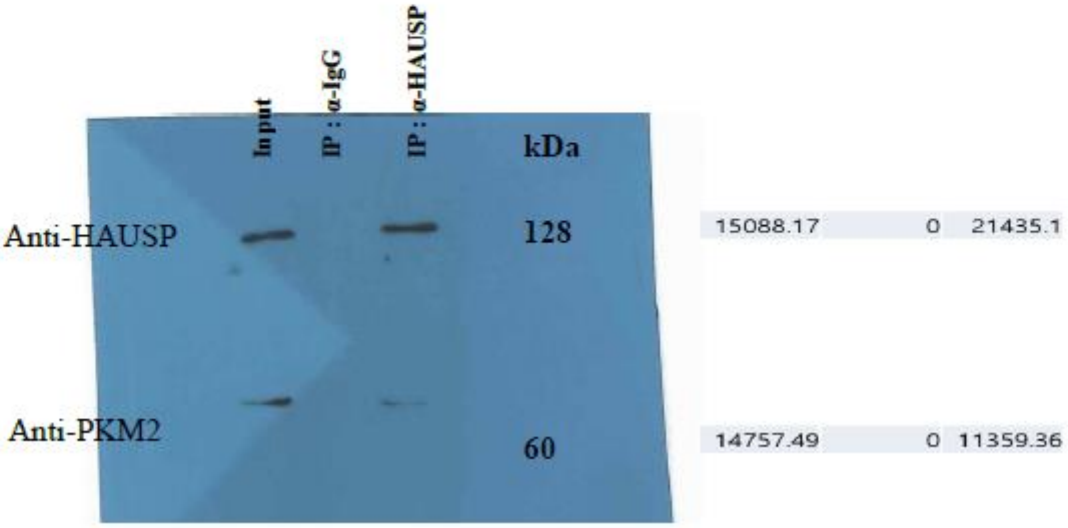
Input
IP : α-IgG
IP : α-HAUSP
kDa
Anti-HAUSP
128
Anti-PKM2
60
15088.17 0 21435.1
14757.49 0 11359.36

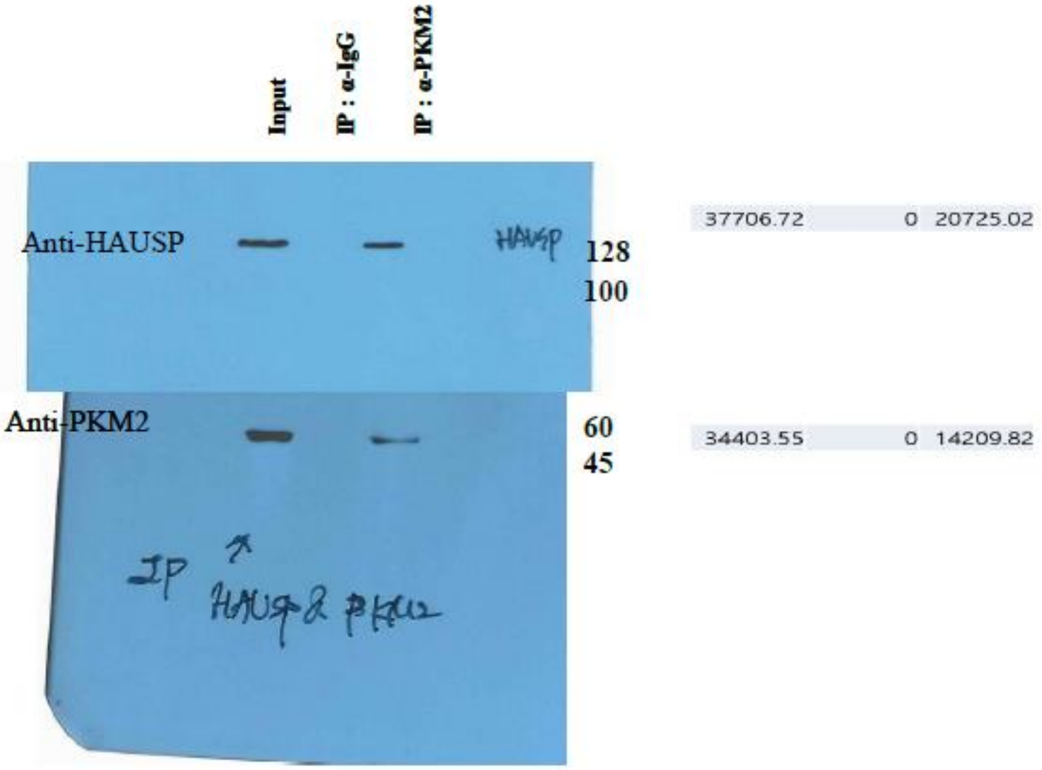
Input
IP : α-IgG
IP : α-PKM2
Anti-HAUSP
128
100
Anti-PKM2
60
45
37706.72 0 20725.02
34403.55 0 14209.82

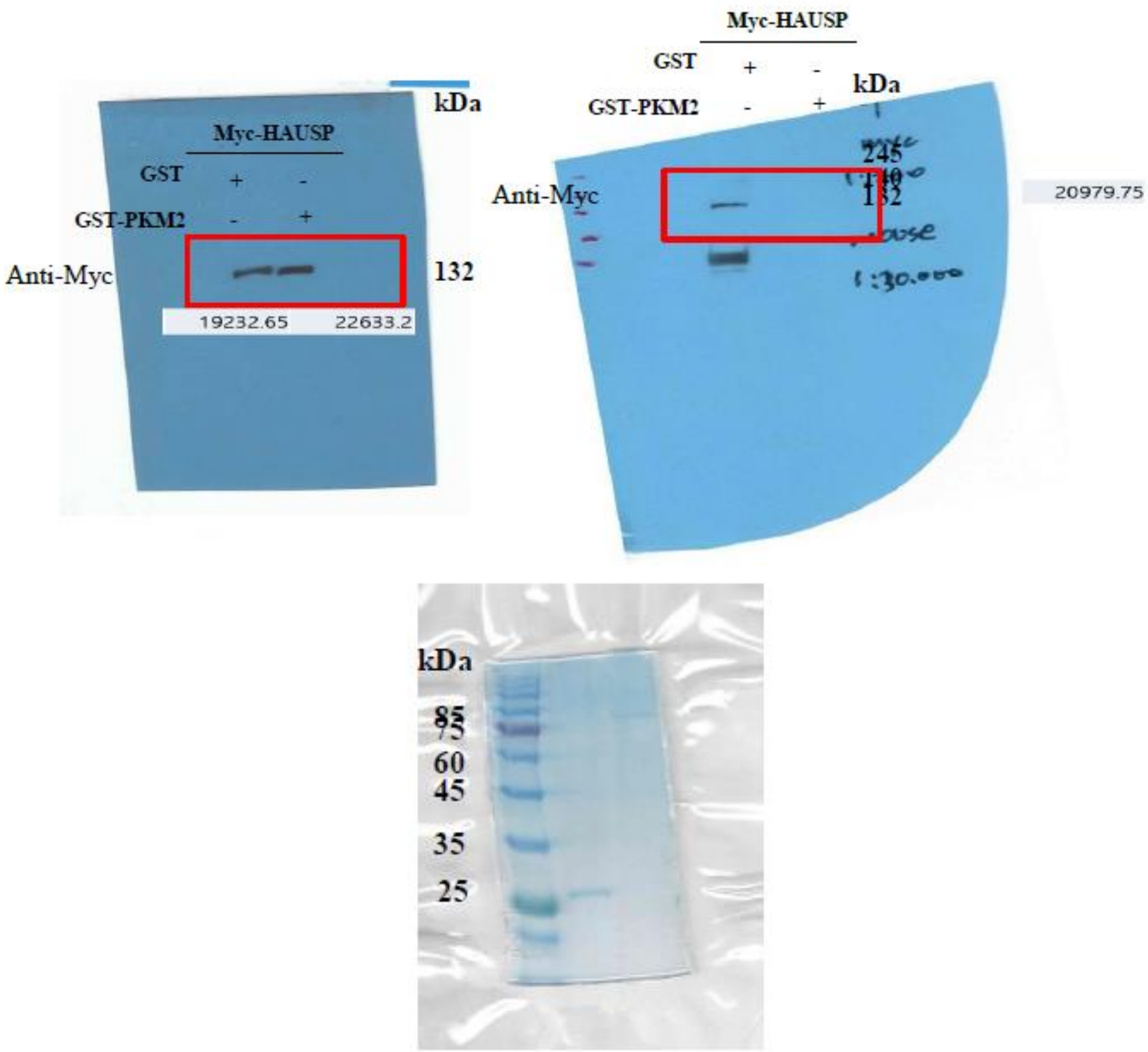

Figure S1. Detailed information about western blot in Figure 1.

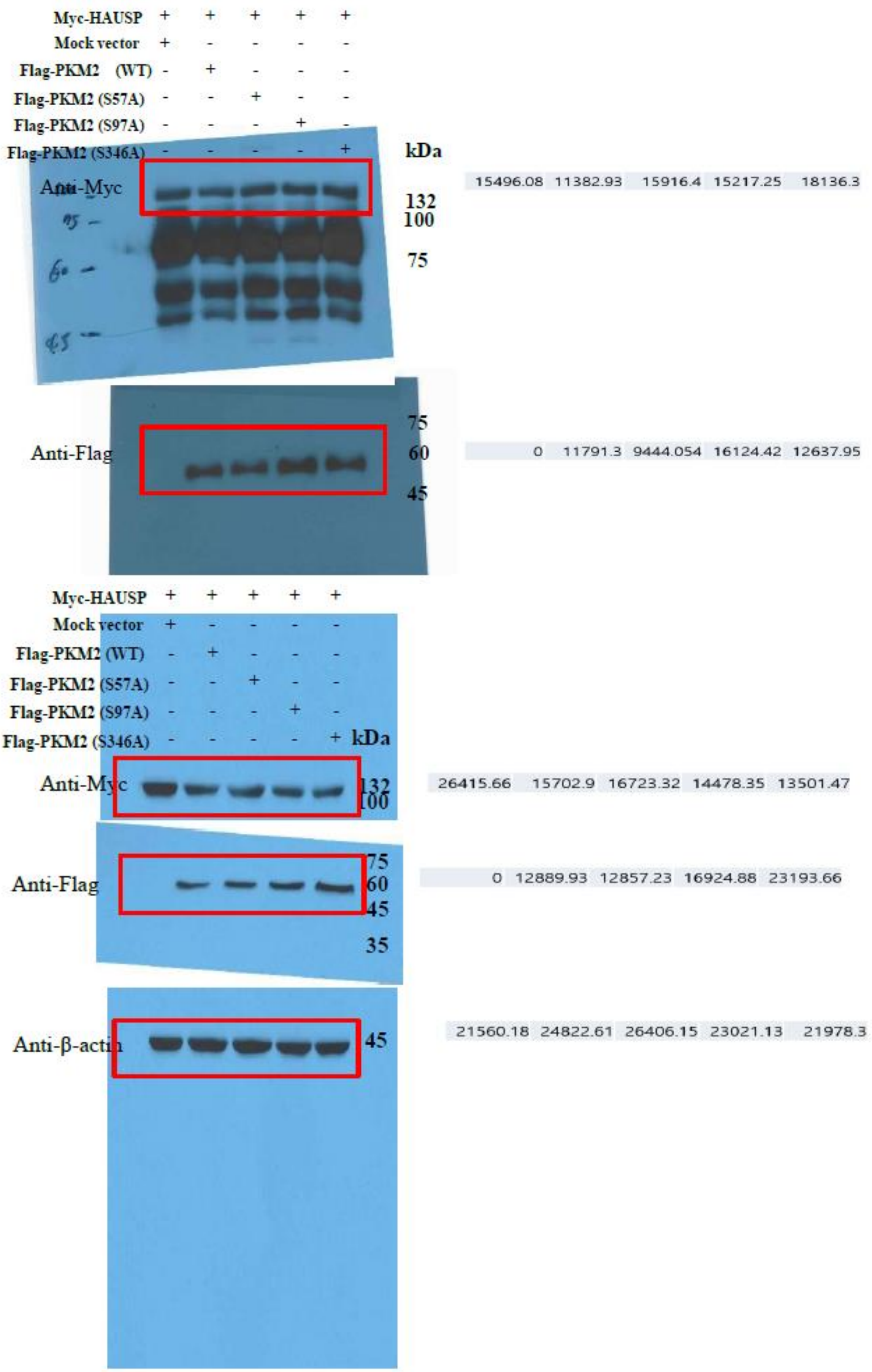

Figure S2. Detailed information about western blot in Figure 2.

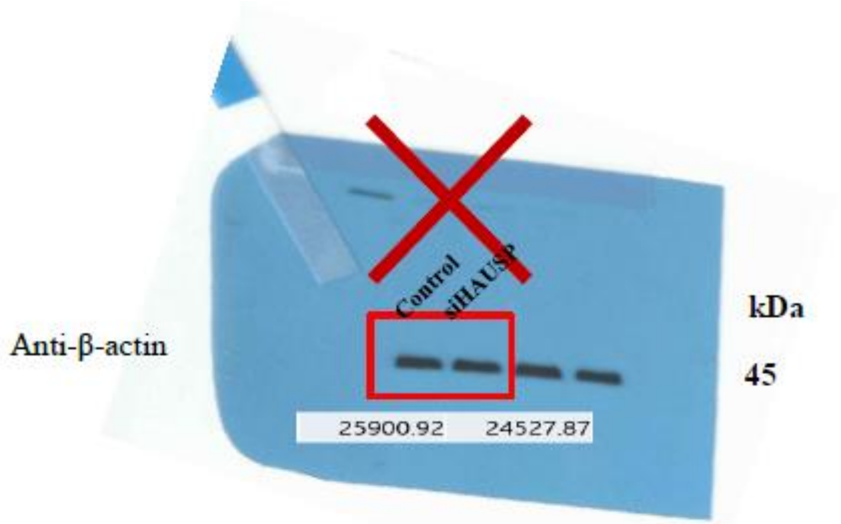
Control
siHAUSP
kDa
Anti-β-actin
45
25900.92
24527.87

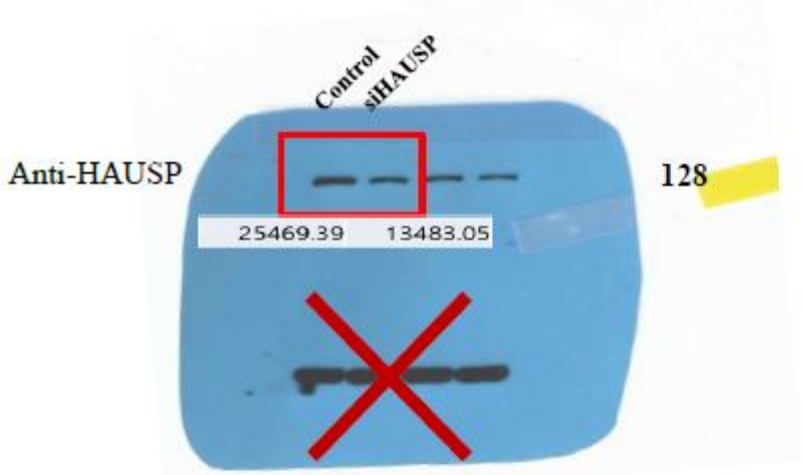
Control
siHAUSP
Anti-HAUSP
128
25469.39
13483.05

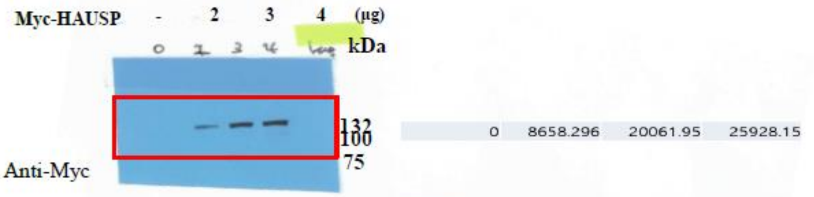
Myc-HAUSP - 2 3 4 (µg)
kDa
132
100
75
Anti-Myc
0
8658.296
20061.95
25928.15

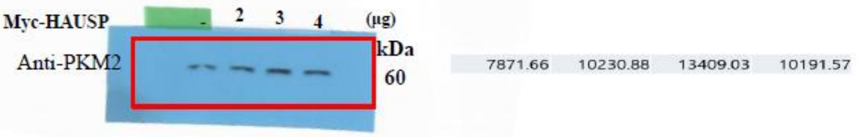
Myc-HAUSP - 2 3 4 (µg)
Anti-PKM2
kDa
60
7871.66
10230.88
13409.03
10191.57

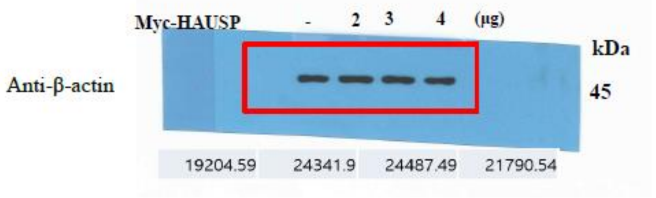
Myc-HAUSP - 2 3 4 (µg)
kDa
Anti-β-actin
45
19204.59
24341.9
24487.49
21790.54

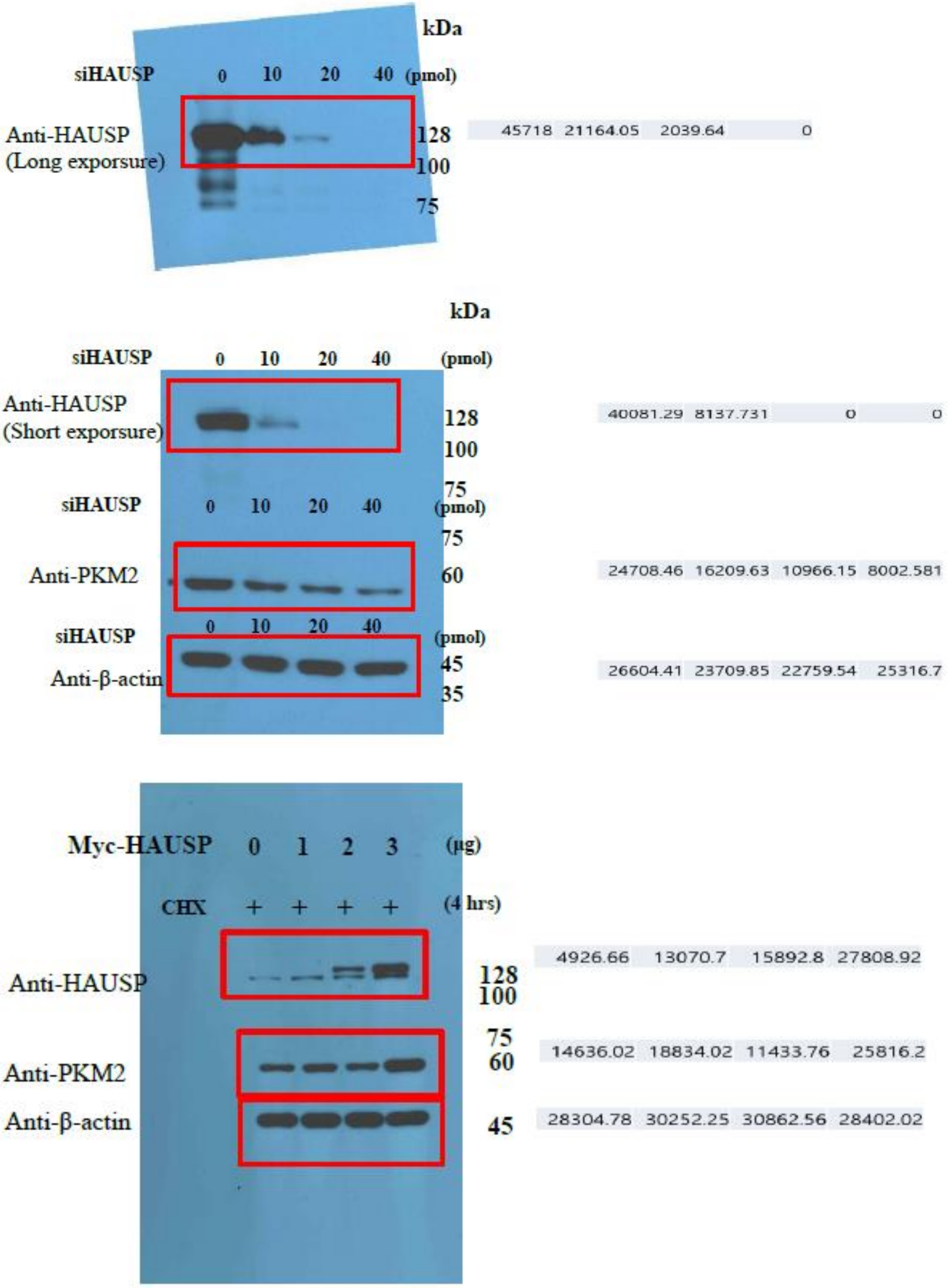

Figure S3. Detailed information about western blot in Figure 3.

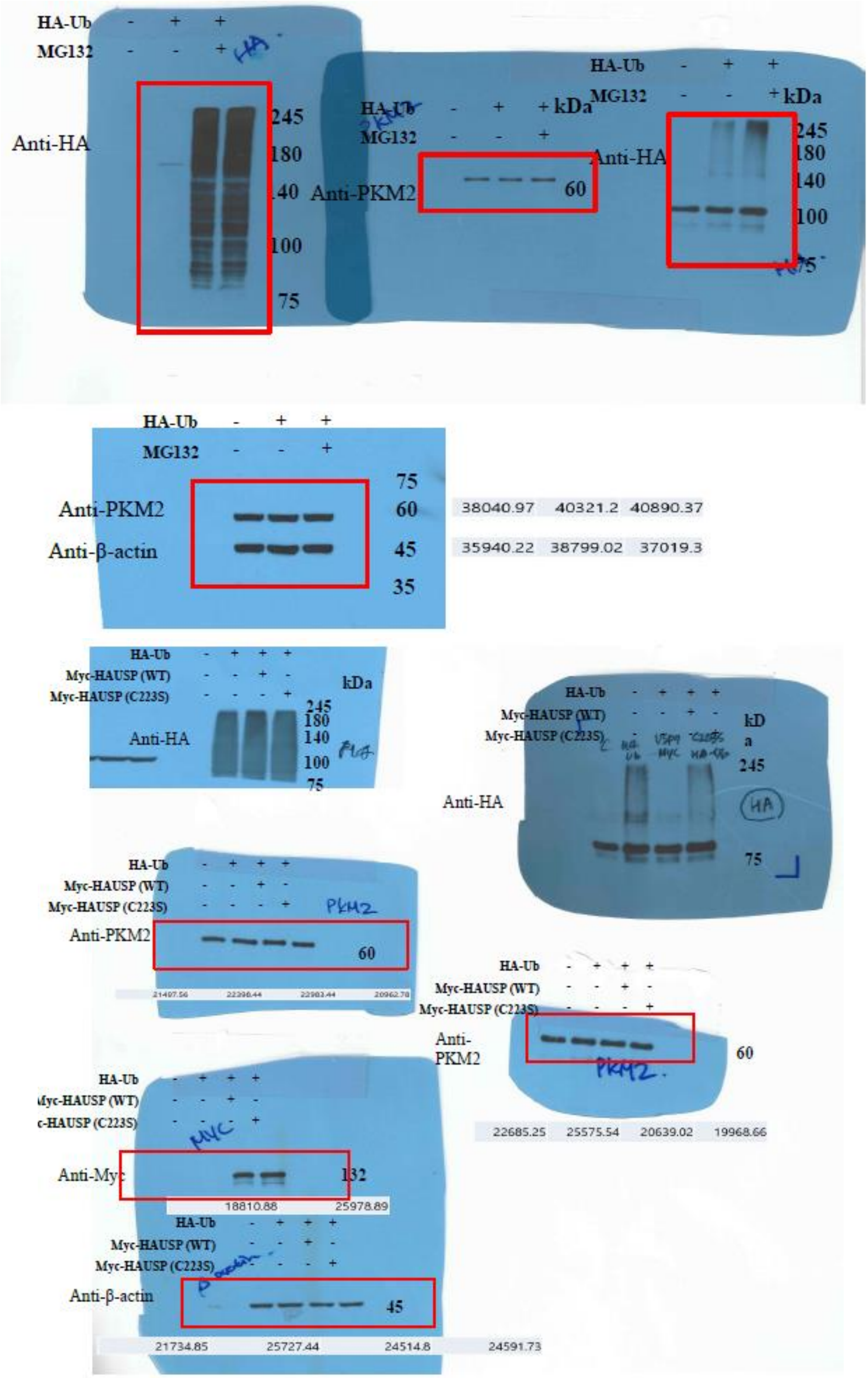
HA-Ub - + +
MG132 - - +
Anti-HA
245
180
140
100
75
HA-Ub - + +
MG132 - - +
kDa
Anti-PKM2
60
HA-Ub - + +
MG132 - - +
kDa
Anti-HA
245
180
140
100
75
HA-Ub - + +
MG132 - - +
Anti-PKM2
Anti-β-actin
75
60
45
35
38040.97 40321.2 40890.37
35940.22 38799.02 37019.3
HA-Ub - + + +
Myc-HAUSP (WT) - - + -
Myc-HAUSP (C223S) - - - +
kDa
Anti-HA
245
180
140
100
75
HA-Ub - + + +
Myc-HAUSP (WT) - - + -
Myc-HAUSP (C223S) - - - +
kD
a
245
Anti-HA
75
HA-Ub - + + +
Myc-HAUSP (WT) - - + -
Myc-HAUSP (C223S) - - - +
Anti-PKM2
60
21497.56 22398.44 22983.44 20962.78
HA-Ub - + + +
Myc-HAUSP (WT) - - + -
Myc-HAUSP (C223S) - - - +
Anti-PKM2
60
22685.25 25575.54 20639.02 19968.66
HA-Ub - + + +
Myc-HAUSP (WT) - - + -
Myc-HAUSP (C223S) - - - +
Anti-Myc
132
18810.88 25978.89
HA-Ub - + + +
Myc-HAUSP (WT) - - + -
Myc-HAUSP (C223S) - - - +
Anti-β-actin
45
21734.85 25727.44 24514.8 24591.73

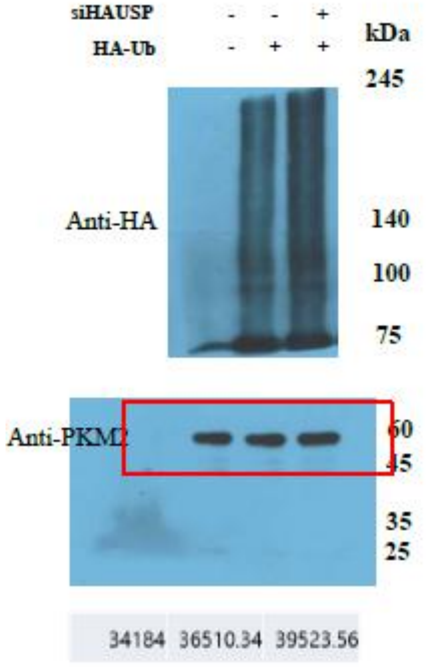
siHAUSP - - +
HA-Ub - + +
kDa
245
Anti-HA
140
100
75
Anti-PKM2
60
45
35
25
34184 36510.34 39523.56

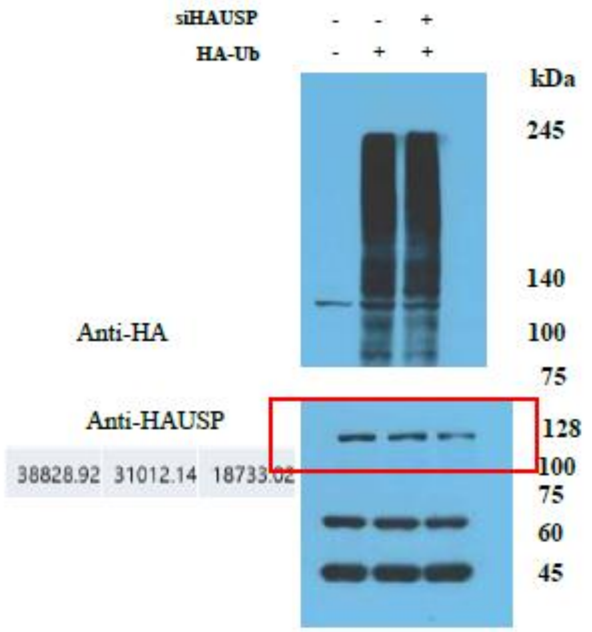
siHAUSP - - +
HA-Ub - + +
kDa
245
140
Anti-HA
100
75
Anti-HAUSP
128
38828.92 31012.14 18733.02
100
75
60
45

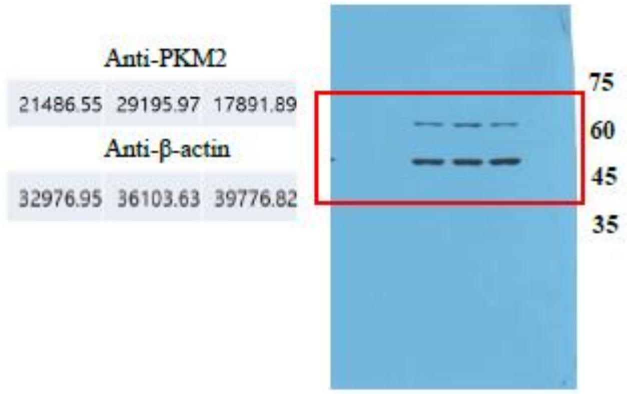
Anti-PKM2
21486.55 29195.97 17891.89
Anti-β-actin
32976.95 36103.63 39776.82
75
60
45
35

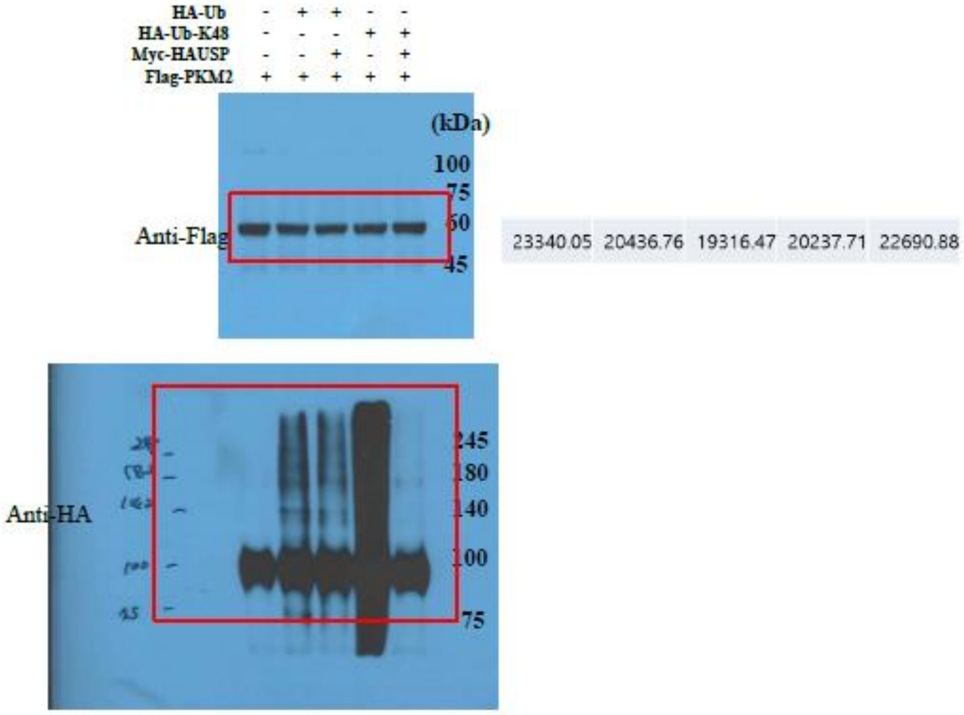
HA-Ub - + + - -
HA-Ub-K48 - - - + +
Myc-HAUSP - - + - +
Flag-PKM2 + + + + +
(kDa)
100
75
Anti-Flag
60
45
23340.05 20436.76 19316.47 20237.71 22690.88
Anti-HA
245
180
140
100
75

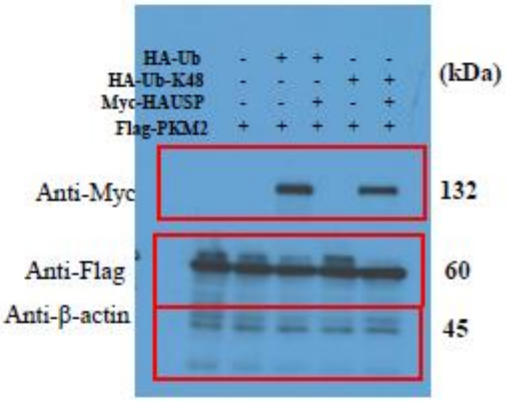

0	0	29175.83	0	22741.37
26762.1	26150.4	24682.88	29617.93	25794.25
20043.42	19330.18	15776.71	14762.35	21478.13

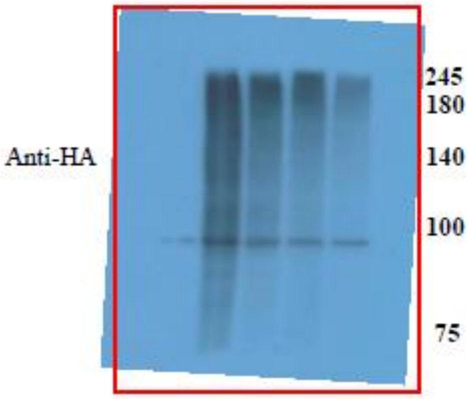

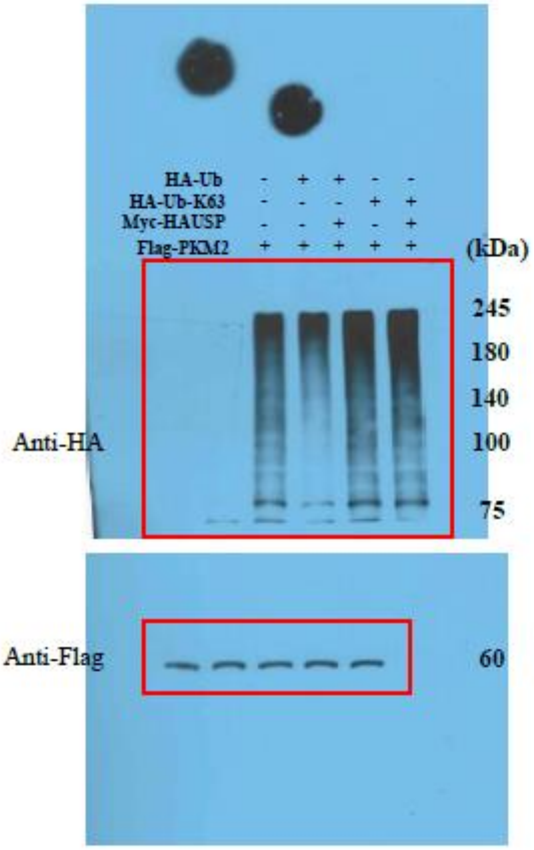

17506.13	15134.15	13367.52	15411.93	15000.81

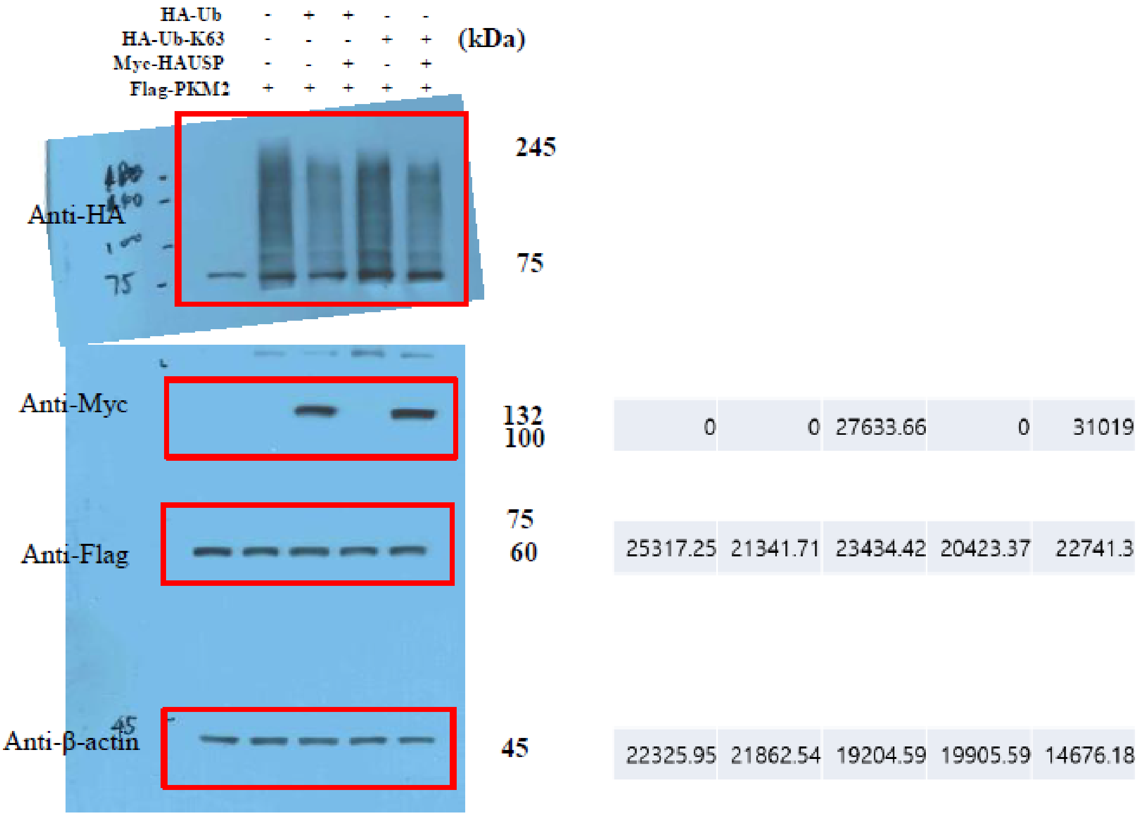

Figure S4. Detailed information about western blot in Figure 4.

© 2020 by the authors. Licensee MDPI, Basel, Switzerland. This article is an open access article distributed under the terms and conditions of the Creative Commons Attribution (CC BY) license (http://creativecommons.org/licenses/by/4.0/).
